# Sexual behaviours, awareness and perceptions towards voluntary medical male circumcision among students in Dr Kenneth Kaunda District, South Africa

**DOI:** 10.4102/HIVMED.v20i1.846

**Published:** 2019-05-22

**Authors:** Sam Mndzebele, Lebogang G. Matonyane

**Affiliations:** 1 School of Public Health, Sefako Makgatho Health Sciences University, Pretoria, South Africa

**Keywords:** Voluntary medical male circumcision, Sexual-behaviours, Awareness, Perceptions, HIV testing

## Abstract

**Background:**

Voluntary Medical Male Circumcision (VMMC) is regarded as the most cost-effective intervention in reducing female-to-male transmission of HIV in countries where heterosexual transmission is the most prevalent mode of infection.

**Objectives:**

The aim of the study was to determine the awareness, sexual behaviours and perceptions of college students in Dr Kenneth Kaunda District, South Africa.

**Method:**

A cross-sectional design was engaged among a sample of 400 students selected using a stratified random sampling method. Descriptive data analysis was engaged to analyse data using STATA 13.

**Results:**

The mean age of the respondents was 23 years. About 50% of the respondents were below the age of 23 years. The majority among the ethnic groups were black people and or African people (87.5%), followed by people of mixed race (8.1%). Most of the students belonged to the Christian religion (94.7%), and about 91.3% were single, while only 6.0% lived with their partners. Among those who were circumcised, a majority (78.0%) had undergone the MMC. About 76.5% of those residing in urban areas, and 80.6% residing in rural areas were circumcised. About 90.3% of the participants had good awareness about VMMC. About 77.3% of the participants disagreed that VMMC reduces the size of the penis, while 57.0% felt that VMMC provides an individual with the status of being a real man in society. Only 14.3% felt that VMMC exposes the penis to environmental hazards. While almost half (47.7%) of the cohort had one sexual partner, about 20.9% had three or more sexual partners.

**Conclusion:**

The findings suggest that there is a high level of awareness on VMMC among college students in relation to its positive role towards reducing STIs and the enhancement of penile hygiene.

## Introduction

Male circumcision (MC) is regarded as one of the oldest and most common surgical procedures performed worldwide. It is undertaken for religious, cultural, social and medical reasons.^1^ Due to the increased use of surgical procedures in the 19th century and advancement in technology within the health system, in the 20th century MC was introduced to some individuals whose culture did not observe male circumcision. This was based on health-related and social reasons. In recent years, voluntary medical male circumcision (VMMC) has evolved. The practice of circumcision commenced in English-speaking countries only in the 19th century; in 2002, only half of the boys were circumcised in the US, whereas other countries such as England and New Zealand had a fairly low circumcision rate.^1,2^ In North Africa and most of West Africa, male circumcision is almost universal; this is in contrast to Southern Africa, which has a low prevalence of circumcision but a high rate of HIV.

A study conducted in Zimbabwe in 2013 found that 50% of the students were willing to go for VMMC, and 50.0% were willing to think about whether to do it or not.^3^ The following year, a similar study indicated that only 0.05% of students were willing to go for circumcision whereas around 50.6% had reservations about undergoing the procedure.^4^ A study among students in the University of Botswana found low circumcision prevalence.^5^ These studies indicate that in countries such as Zimbabwe and Botswana, there are still some young men who have not been circumcised. In the South African context, there is reportedly moderate prevalence of circumcision among students in higher institutions of learning as indicated by the survey done in 2014 by Higher Education and Training HIV/AIDS Programme (HEAIDS), which showed that 66.0% of the students were circumcised, 79.0% preferred VMMC and the students were willing to encourage others to opt for circumcision.^6^ This prevalence in South Africa is because of massive scale-up of VMMC in different communities and mobilisation through media platforms targeting young people.

In December 2014, over 9 million VMMCs were performed in all of the 14 priority countries in sub-Saharan Africa.^7^ In 2014, only 3 million VMMCs were performed, which showed that rapid VMMC scale-up has been implemented in most of sub-Saharan Africa. According to UNAIDS targets, it is expected that by 2020, there should be an additional 27 million VMMCs done; these VMMC targets were based on the statistics obtained in 2014.^7^ There has been significant progress made in scaling up VMMCs in 14 priority countries; during 2015 Ethiopia and Kenya exceeded their 80.0% coverage of men performing VMMCs; Tanzania was on track to reach the expected target. Countries such as Lesotho, Malawi, Rwanda and Zimbabwe were still lagging in 2015 with a low coverage of 6.0% – 26.0%. There is also an urgent need to launch VMMC in Central African Republic and South Sudan.^7^

The Voluntary Medical Male Circumcision programme was launched in Kenya in 2008 following the WHO/UNAID recommendation in 2007. Initially, Kenya aimed to circumcise 860 000 by 2013. The number of operations increased every year from 8000 to 190 000 between 2008 and 2013. According to literature, 80.0% of the VMMCs in Kenya were conducted in the Nyanza province, where half of the uncircumcised men live in Kenya.^8,9^ There is evidence of the overall success of the VMMC programme in Kenya, as most young people begin their adolescent state to acknowledge this and ensure they have done VMMC; the Kenyan government seeks to ensure that there is sustainability of the programme.^10^ According to the Centers for Disease Control and Prevention (CDC), the next step in Kenya is to accelerate VMMC among males under the age of 15 and infants.^11^ By the end of 2014, in Lesotho there were 85 000 men who had VMMC as part of HIV comprehensive prevention services; 56.0% of those men were tested for HIV; while in South Africa between 2012 and 2015 250 000 circumcisions were done.^7,12^

It is now well documented that a significant portion of South Africa’s indigenous population has been practicing the culture of male circumcision for centuries. For instance, male circumcision initiation schools in South Africa form part of the cultural practice. However, the integration of medical male circumcision with traditional manhood initiation rituals still seems to lack acceptability in these societies. Of note, about 70.0% of the men fear being stigmatised if they are circumcised medically in South Africa.^13^ As part of VMMC promotion and awareness in South Africa, a number of organisations have been working closely with HEAIDS to assist with comprehensive HIV prevention services by motivating all male students to get circumcised. Within the institutions of higher learning, such as colleges, there are health promoters and VMMC mobilisers who continuously interact with students about the implications that can be endured from having unsafe sex and the benefits of VMMC. According to statistics, young people aged 15–24 account for 39.0% of new HIV infections, which is a startling proportion.^14^ A study conducted in Uganda among fishing communities reported that there are several cultural beliefs about VMMC, which include fear that foreskins are sold after removal, the belief that a VMMC recipient’s first sexual partner after the procedure should not be his spouse, and the belief that vaginal fluids aid circumcision wound healing. On that note, some researchers state that the primary motivations of VMMC are religious injunction, hygiene and protection against sexually transmitted infections (STIs) (not necessarily HIV).^15,16^ A study conducted in Kenya suggested that there is a need to dispel misconceptions about MC by involving religious leaders and women’s groups, and making circumcision relevant to men who are already practicing an HIV preventative method. Another study conducted among university students in Zimbabwe revealed that only a minority of them were willing to be circumcised; this was mainly due to the negative attitude that students had about VMMC.^4,17^

A cross-sectional study was also conducted among adults in rural Uganda, which revealed different negative perceptions about VMMC, especially in relation to its HIV protective measure. There is a concern about circumcision that is done after childhood. A study conducted among police officers in Dar es Salaam, Tanzania, raised concerns about circumcision conducted at older ages, as there is a view that pain can be felt more and penile erection post circumcision can delay wound healing.^18,19^ The integration of male circumcision into mother and child health services can motivate women to allow circumcision of their child at an early age, hence alleviating many negative beliefs that the child can have as they grow older when they are not circumcised. Furthermore, a group of researchers indicated that if more health education can be provided about different aspects of VMMC, some of the perceptions can be eliminated and corrected. Studies conducted in most African countries indicate that there is a lack of knowledge and awareness about VMMC; while another study that was conducted among university students in Botswana revealed varied evidence that there is an increase in the level of awareness of VMMC.^5,20^ Similarly, a study conducted among university students in Zimbabwe found that the majority of students were aware about VMMC but failed to provide detailed information about the procedure. Despite people being educated through various media platforms and promotion campaigns, literature has revealed that there is a knowledge gap about certain aspects of VMMC.^3,18^ A study conducted in Zimbabwe indicated that exposure to information on VMMC is important in order to increase the awareness. The most cited sources of information were radio, television, newspaper and billboards. Another study conducted among university students in Zimbabwe showed that the majority of students were aware of VMMC but failed to provide detailed information about the procedure; the only information they had was from public media.^3,17^ There have been varied views by the general population regarding VMMC since its inception. For instance, there is still a strong belief in southern Africa that circumcision decreases natural sexual ability.^5^

Within the context of eastern and southern Africa, where HIV prevalence is high, mathematical modelling has shown that circumcising 80.0% of males (aged 15–49) in five years can prevent approximately 3.4 million infections. There has been a belief that post VMMC, sexual behaviours, especially of males, can potentially reduce the expected benefits of the practice.^18^ A demographic and health survey (DHS), done from 2010 to 2011 in Zimbabwe, found no association between MC status and risky sexual behaviour.^17^ Recent studies conducted in Zimbabwe and Uganda provided varied evidence that MC is sometimes viewed as a complete protection against HIV and STIs. Some observational studies conducted in the field of VMMC have found that men who are circumcised are more likely to engage in risky sexual behaviour than those who are uncircumcised.^15,17,20^ In Africa there is a strong belief that circumcision improves sexual performance, especially among adults, and there is also a concern that this can increase the risk of HIV transmission. Equally so, there is a strong believe that the perceptions that communities hold about VMMC can likely affect their sexual behaviours after undergoing the procedure.^18,19^ A study conducted among men in western Kenya revealed that most men felt that condoms are much easier to use after VMMC as indicated by messages they got during circumcision.^21^

There is a very strong association between being circumcised and risky sexual behaviour; however, despite the association, men who are circumcised are less likely than those who are uncircumcised to be HIV-positive; this clearly indicates that the benefits of being circumcised outweigh the risk of predicted unwanted sexual behaviour.^22^ In a study done to examine sexual behaviour change following circumcision among adult men in Siaya and Bond District hospitals in western Kenya, a cohort of men who chose to be circumcised were matched with those who chose not to be circumcised; in the three months of the study, risky sexual behaviour was reported among the circumcised cohort but at the 12 months follow-up there were no significant differences in risky behaviour.^1^ This kind of study demonstrates that if proper counselling is offered to men on risk reduction, they are more likely not to engage in risky sexual behaviour following circumcision. The study aimed at determining the awareness, sexual behaviours and perceptions of college students in Dr. Kenneth Kaunda District in South Africa.

## Methods

### Study population and sampling method

Through the use of a cross-sectional design, a population comprising male students between the ages of 18 and 49 years were registered at Vuselela College. There were estimated 4000 male students registered in 2015 as per the Department of Higher Education report. Each campus was estimated to have the following number of male students enrolled: Potchefstroom Centre for Information Communication and Technology Studies (800), Jourbeton Centre for Engineering Studies (1800), Klerksdorp Centre for Business Studies (1200) and Matlosana Campus (200). Hence, a stratified random sampling method was found suitable, where each campus was stratified based on the number of male students enrolled. The resultant sample size arrived at was 351 participants, which was increased with a buffer of 15.0% to 400.

### Data collection instrument and pretesting

A self-administered questionnaire was used as a data collection tool. The questionnaire comprised closed-ended questions. Questions were developed in English, as students were in a higher institute of learning. Data were collected until a required sample reached. The data collection instrument comprised four sections, which included socio-demographic characteristics, awareness of VMMC, sexual behaviours and perception towards VMMC. Good and/or satisfactory awareness, perceptions and practices regarding VMMC were determined through positive or preferred responses to > 75% of the questions in the questionnaire as indicated by the codes. The questionnaire was pretested among 15 students from Springfield College, who do not form part of the study sample.

### Data collection process

After proper arrangements with the lecturers and college authority, students were approached in classes and briefed about the study. Those selected to take part in the study were asked to remain in class to be informed more about the study. Those who agreed to participate were asked to sign an informed consent form with detailed information about the purpose of the study and potential risks, such as being asked certain questions that they were comfortable to answer. Participants were therefore provided with the questionnaire and instructions on how to complete it. After completion, questionnaires were collected by the researcher. The process took them 20–25 min.

### Validity and reliability

The reliability of the data collection tool was enhanced by conducting a pilot study among a few students. Sample size was increased by a marginal number to cater for non-responses and incomplete questionnaires. Questions were carefully developed in such a way that they address the study objectives, hence ensuring the reliability of the data collection tool. Questionnaires were checked by the supervisor to ascertain if they are relevant to the study. To ensure validity of the data collection instrument, questions were structured using simple language that participants understood. Questions were simple and explained clearly the aim of the study and its importance, thus minimising non-response. To minimise selection bias, the researcher used a stratified random sampling method, which involved the division of a population into smaller groups known as strata. Unavoidable information bias, more specifically recall bias, was expected to be encountered; however, this was borne in mind when interpreting the results of the study.

### Data capture and analysis

The completed questionnaires were cross-checked to remove mistakes. Data were later entered into an Excel spreadsheet and coded, then finally imported into STATA 13 (STATA Corp, TX, USA) for statistical processing and analysis. Univariate analyses, including frequency distributions, were run to characterise the sample. Bivariate analysis was done to obtain significant association between the awareness and perception of college students in relation to their sexual practice. The chi-square test and odds ratios (OR) with 95.0% confidence intervals were used to measure the association. Summary measures were used to describe findings, and they were expressed as means (standard deviations), medians (ranges), modes and proportions.

## Ethical consideration

Permission to conduct the study was granted by Sefako Makgatho Health Sciences University School of Healthcare Sciences Research Ethics Committee with clearance certificate Ref. [SMUREC/H/1112017: PG]. Furthermore, approval was granted by the Provincial Department of Higher Education and Training (DHET), as well as the Dean of Vuselela College in order to gain access at the institution for data collection. Participants were first clearly briefed about the purpose of the study prior to obtaining an informed consent. Further, participants were informed that participation in the study was voluntary and that they were free to withdraw from the study at any time without being penalized. Participants were also assured of anonymity in the completion of the questionnaires through the use of codes and not their real names or personal details. During the process of the study, confidentiality was ensured and maintained by not sharing participants’ information with anyone, and by keeping all the study material in a secure place.

## Results

### Sample characteristics

The mean age of the respondents was 23 years, with a minimum of 18 and a maximum of 42 years of age and a standard deviation of 4.1. About 50.0% of the respondents were below 23 years. Those below 23 years were 59.5% (*n* = 191) as compared to those above 23 years (*n* = 130; 40.5%). The majority ethnic group in the study was Black people or Africans (87.5%), followed by Mixed race (8.1%). Most students belonged to the Christian religion (94.7%), and there were also Jewish (1.87%) as well as Muslim (1.87%) students. A majority of the students were single (91.3%), with around 19 (6%) living with their partners and 187 (*n* = 187; 58.3%) participants residing in urban areas. About 47.7% of the participants reported one sexual partner, and 35.5% reported two or more sexual partners. Of note, students who had undergone circumcision were more likely to have one sexual partner (53.3%).

### Uptake of male circumcision and sexual behaviours

The proportion of circumcised men in the cohort was 77.6%. Among those circumcised, the majority (78.2%) had undertaken the medical male circumcision; the rest underwent traditional male circumcision. One reason provided by the participants for not being circumcised included fear of complications (21.1%), while about 45.1% had no specific reason for not being circumcised. About 51.8% had a single partner and was circumcised, with 22.3% having three or more partners. Participants who resided in the urban areas were mostly circumcised (76.5%), those who were uncircumcised from urban areas were only 23.5%. Those who resided in rural areas and were not circumcised constituted 19.4% compared to those who were circumcised (80.6%). A significantly high proportion of the participants rightly acknowledged health benefits of undergoing VMMC as revealed in the following percentages: 82.2% indicated that VMMC increases penile hygiene; 95.6% reported that VMMC reduces the risk of STIs; and 92.2% said that VMMC reduces the risk of penile cancer. In terms of sexual behaviours, almost half (47.7%) of the cohort had one sexual partner; about 20.9% had three or more sexual partners.

### Level of awareness

The majority of the participants (90.3%) had good awareness about issues surrounding VMMC. Participants below the age of 23 years (54.8%) had a poor awareness level. Specifically, the majority of participants (97.8%) were aware that VMMC involves the removal of the foreskin. In terms of penile hygiene knowledge regarding VMMC, most participants (82.2%) scored well on this aspect. A very high number of the participants (95.6%) had a strong belief that VMMC reduces the risk of STIs. Participants who knew that there is a need for abstinence for six weeks after VMMC were about 91.9%. About 30.6% of the respondents reported that they have learnt about VMMC on television, while only a few (1.3%) reported that they have never learnt about VMMC at all. Those who learnt from the radio were about 18.4%, from newspaper 15.6%, from clinic and/or hospital 38.3%, from friend 27.1%, from neighbours 10.0%, through posters 13.7%, and from other sources 8.1%.

### Perception of medical male circumcision

Generally, only 10.6% of the participants had positive perceptions about VMMC. In terms of the following statements: ‘VMMC reduces the size of the penis’, and ‘VMMC decreases sexual satisfaction’; 77.26% of the participants disagreed with the former and 75.63% with the latter. When asked whether circumcised men enjoy sex more than uncircumcised men, about 61.99% agreed with this statement. In terms of whether women prefer circumcised sexual partners to uncircumcised partners, about 58.57% agreed. Further, about 54.2% disagreed that VMMC violates the principles of traditional male circumcision (as indicated in Table 1).

**TABLE 1:** Participants’ perceptions towards voluntary medical male circumcision.

Perception	Agree		Disagree		Don’t know	
	*n*	%	*n*	%	*n*	%
Medical male circumcision reduces the size of the penis	15	4.67	248	77.26	58	18.07
Medical male circumcision decreases sexual satisfaction	32	10.00	242	75.63	46	14.38
Medical male circumcision proves that an individual is a real man	84	26.17	183	57.01	54	16.82
Women prefer circumcised sexual partners to uncircumcised partners	188	58.57	59	18.38	74	23.05
Medical male circumcision violates the principles of traditional male circumcision	54	16.82	174	54.21	93	28.97
Medical male circumcision makes the penis more vulnerable to environmental hazards	46	14.33	170	52.96	105	32.71
Circumcised men enjoy sex more than uncircumcised men	199	61.99	51	15.89	71	22.12

## Discussion

### Uptake and barriers towards voluntary medical male circumcision

In our study, a significantly higher (78.2%) proportion of participants had undergone VMMC. This figure is above the one recorded by HEAIDS in Southern Africa in 2014, which indicated that about 66.0% of the students were circumcised, bearing in mind that our study was conducted three years later. These developments may also be an indication of a rapid scale-up of VMMC promotion and awareness by non-governmental organisations (NGOs) targeting young people within the Dr. Kenneth Kaunda District. On another note, around 21.1% participants in our study reported fear of complications, and 16.1% said they fear pain following VMMC. These findings are consistent with findings in most literature of these barriers associated with VMMC. For instance, these barriers (fear of pain, concerns around safety issues, and the costs) were mentioned by participants in related studies.^1,20,23,24,25^ Our findings indicated that about 47.7% of the participants reported one sexual partner, and 35.5% had two or more sexual partners. Of note, students who had undergone circumcision were more likely to have one sexual partner (53.3%). This is an indication that there was a limited presence of risky sexual behaviour among the participants in our study.

### Voluntary medical male circumcision awareness among students

The high proportion (90.3%) in terms of good awareness on VMMC issues among students in our study seems to be in line with the findings of a study conducted among university students in Botswana, where they found that 95.4% had good knowledge about VMMC.^5^ In terms of VMMC’s protective role against HIV, we noted that participants lacked knowledge (30.8%) on this aspect. This is consistent with related findings in studies, such as those in Botswana and Uganda, whose findings revealed that there was still a knowledge gap among students about such aspects regarding sexual matters and VMMC.^5,18^ In contrast, Engle and others conducted a study in Kenya, which showed increased awareness of VMMC among participants, especially in relation to its role in partial HIV protection.^21^ In our study, only 18.4% of the participants claimed that they learnt about VMMC from the radio; while about 15.6% learnt from newspapers. About 38.3% learnt about VMMC from clinics/hospitals, and 27.1% and 13.7% from friends and posters, respectively. We also noted that these findings are similar to a study conducted in Zimbabwe, which indicated almost the same sources of information to their participants such as radio, television, newspaper and billboards.^17^

### Some health benefits associated with voluntary medical male circumcision

There are certain health benefits associated with being circumcised, which include the reduced risk of acquiring HIV, cancer, and STIs, in addition to an increased level of penile hygiene. In all of these aspects, a significantly high proportion of the participants in our study rightly acknowledged these benefits in the following percentages: 82.2% indicated that VMMC increases penile hygiene; 95.6% reported that VMMC reduces the risk of STIs; and 92.2% said that VMMC reduces the risk of penile cancer. These ratings are closely similar to other findings conducted in a number of studies within the African region in terms of VMMC and its health benefits such as in Botswana;^26^ South Africa;^24,27,28^ Zimbabwe;^29^ and Kenya.^23^

### Perception regarding voluntary medical male circumcision

In terms of perception towards VMMC, our study results revealed that only 14.3% of the participants reported that VMMC makes the penis more vulnerable to environmental hazards, while only 10.0% reported that VMMC decreases sexual satisfaction. Such perceptions from students are consistent with some studies conducted among university students in Zimbabwe, which revealed that only a minority of them were willing to be circumcised.^4^ Further, a cross-sectional study that was conducted among adults in rural Uganda revealed some negative perceptions towards VMMC.^18^ However, there is evidence that such negative perceptions towards VMMC may be minimised in societies through the involvement of community leaders and religious groups in aspects of VMMC education and promotion. Our study also revealed that students who were circumcised were more likely to have a positive perception towards VMMC than those who were uncircumcised at 91.8% and 8.2%, respectively.

## Study limitations

The study was limited to students in the selected colleges only; there were no other young people from the society included in the study to increase external validity. The study was relying mostly on self-reported information regarding the circumcision status of the participants. There was no physical examination done to confirm the circumcision status. Finally, like in most similar studies, there might be some recall bias as some participants may not accurately remember where they have learnt and/or heard about circumcision.
